# Exclusion of Estrogenic and Androgenic Steroid Hormones from Municipal Membrane Bioreactor Wastewater Using UF/NF/RO Membranes for Water Reuse Application

**DOI:** 10.3390/membranes10030037

**Published:** 2020-02-27

**Authors:** Mujahid Aziz, Tunde Ojumu

**Affiliations:** Faculty of Engineering and the Built Environment, Department of Chemical Engineering, Cape Peninsula University of Technology, Bellville, Cape Town 7435, South Africa; OjumuT@cput.ac.za

**Keywords:** membrane bioreactor (MBR), ultrafiltration (UF), nanofiltration (NF), reverse osmosis (RO), androgen, estrogen, steroid hormones, level of detection (LOD), micro pollutions (MPs), predicted no-effect concentration (PNEC)

## Abstract

In the context of water scarcity, domestic secondary effluent reuse may be an option as a reliable source for alleviating acute water shortage. The increasing risks linked with the presence of natural steroid hormones and many emerging anthropogenic micropollutants (MPs) passing through municipal wastewater treatment works (MWWTWs) are of concern for their endocrine-disrupting activities. In this study, domestic wastewater treated by a full-scale membrane bioreactor (MBR) at an MWWTW in the Western Cape Province, South Africa, was used directly as the influent to a reverse osmosis (RO) pilot plant for the removal of selected natural steroid hormones 17β-estradiol (E_2_) and testosterone (T) as a potential indirect water recycling application. Estrogenicity and androgenicity were assessed using the enzyme-linked immunosorbent assays (ELISA) and the recombinant yeast estrogen receptor binding assays (YES). The influent pH and flux did not influence the rejection of E_2_ and T, which was most likely due to adsorption, size exclusion, and diffusion simultaneously. RO and nanofiltration (NF) exhibited excellent removal rates (>95%) for E_2_ and T. All the E_2_ effluent samples with MBR/ultrafiltration (UF), MBR/NF, and MBR/RO were lower than the US EPA and WHO trigger value of 0.7 ng/L, as well as the predicted no-effect concentration (PNEC) values for fish (1 ng E_2_/L).

## 1. Introduction

The growing fear of a shortage of water resources is becoming an important topic as a severe paucity of water has been seen all over the globe. There is an increasing concern about the potentially harmful effects of some substances present in water bodies. These emerging micropollutants (MPs) have been shown to be present in both industrial and domestic wastewater in unnoticeable quantities, with concentrations ranging from micro- and nanograms per litre scales [1]. Several natural and human-made compounds have been shown to modulate endocrine activity in vertebrates. Compounds acting in this way are collectively referred to as endocrine-disrupting chemicals (EDCs). These chemicals enter the sewer system through disposal or excretion and are not completely removed during wastewater treatment. Numerous studies have detected EDCs, such as natural steroidal hormones [2] and pharmaceuticals and personal care products (PPCPs) in environmental samples. Studies have shown antagonistic effects on aquatic wildlife [3] that have been linked to the presence of EDCs and PPCPs. These natural hormones are responsible for maintenance, reproduction, development, and behaviour of organisms [4]. Among the sources of these substances are natural steroid hormones, industrial chemicals, pharmaceuticals, and many others [5]. Many researchers have investigated the effect of these substances in water bodies, observing harmful effects on humans and animals, such as endocrine system anomalies, cancer, reduction of sperm quantity, and endometriosis [6]. Authors have confirmed decreased testosterone levels heightened anxiety [4] The natural steroid estrogens, estrone (E_1_), 17β-estradiol (E_2_), and estriol (E_3_), and the synthetic 17α-ethinylestradiol (EE_2_), are the most widely investigated because of their high estrogenicity at low concentrations and their presence in several matrixes, such as drinking, ground, and surface water, as well as effluents from municipal wastewater treatment works (MWWTW) [7]. E_1_, E_2_, and E_3_ are primarily female hormones. The most potent estrogen, 17β-estradiol (E_2_), and its precursor, T, play critical roles in mammalian reproductive processes. Evidence indicates that these steroids are present in a bioactive form in the excretions of many male mammals [8]. The natural androgen, T, and the natural estrogen, E_2_, will end up in the environment through sewage discharge and animal waste disposal [9].

The treated sewage effluent and natural surface water mixture found in the City of Cape Town (CoCT) rivers are used directly for irrigation in the agricultural areas. The Western Cape Province, South Africa, has a high rainfall in winter with very low or no rain in summer. During the summer months, most of the water in these rivers is treated sewage effluent [10]. Thus, estrogens and androgens are not completely removed by MWWTWs, justifying this research to find more efficient processes to remove these intractable pollutants. Given that conventional treatment processes are less efficient, the scientific community set out in search of novel processes and operating conditions, which could increase the treatment efficiency of wastewater [11]. Nanofiltration (NF) and reverse osmosis (RO) membrane filtration processes have also been used to produce high-quality water from non-traditional sources such as brackish, seawater, or secondary treated wastewater [12]. Research has demonstrated the excellent capacity of NF/RO to remove a large range of MPs in pilot and full-scale applications [13].

Municipal wastewaters are mostly treated by conventional activated sludge (CAS) or membrane bioreactor (MBR) processes. The MBR process is simply an integrated treatment system of microfiltration (MF) or ultrafiltration (UF) membranes with a biological reactor where secondary effluents often include high concentrations of dissolved matter, pesticides, pathogen, heavy metals, and micropollutants [14], thus making tertiary treatment a necessity for potential water reuse. Although tertiary processes play an essential role and can be applied after the secondary process at MWWTPs, there is still a lack of information about these processes and their EDC removal capacity from environmental matrixes at trace concentration [15]. Over the last decades, the use of membrane technology has grown considerably in wastewater treatment. It has proven to be an effective method for the removal of a wide variety of contaminants from wastewater. Some studies concluded that an integrated system of MBR-NF/RO could be considered as a good alternative for the recovery and reuse of treated wastewater for irrigation [16,17,18].

Total estrogenicity of a sample is commonly measured to avoid exhaustive chemical analysis in chemically complex samples, including wastewaters [19]. Enzyme-linked immunosorbent assay (ELISA) and yeast estrogen screen (YES) bio screening assays are strong, rapid, simple, and cost-effective methods for quantitative analysis of estrogenic hormones, such as E_2_ and T [10]. These in vitro bioassays play a crucial role for the ecotoxicological assessment of water and wastewater quality because they determine the joint toxicity caused by complex samples, often regarding a specific mode of action [20].

In 2018, the City of Cape Town (CoCT) suffered a third consecutive year of severe drought due to unpredictable weather patterns resulting in a significant shortage of water in the Western Cape Province, South Africa. Water reuse may be an option to alleviate acute water shortages if appropriate treatment technologies can be developed. This study aimed to demonstrate the complementarities of combining a RO pilot plant with a full-scale MBR at a MWWTW while investigating the concentration and removal efficiencies of an estrogen (17β-estradiol) and an androgen (testosterone) steroid hormone, detected in the influent and effluents with UF, NF, and RO membranes for potential indirect potable water reuse.

## 2. Materials and Methods

### 2.1. Full-Scale MBR

The WWTW with a full-scale MBR plant is located in the Western Cape and receives its wastewater from the largest informal settlement in the province. Raw sewage from the WWTW served as the feed, after filtration, for the MBR. The MBR output was filtered in a membrane tank by a commercially available, plate and frame type, hollow fibre, submerged UF membrane. The MBR system incorporates ZeeWeed 500 ultrafiltration membranes (GE Zenon), producing 18 megalitres of effluent per day.

### 2.2. RO Pilot Plant

**Figure 1:**
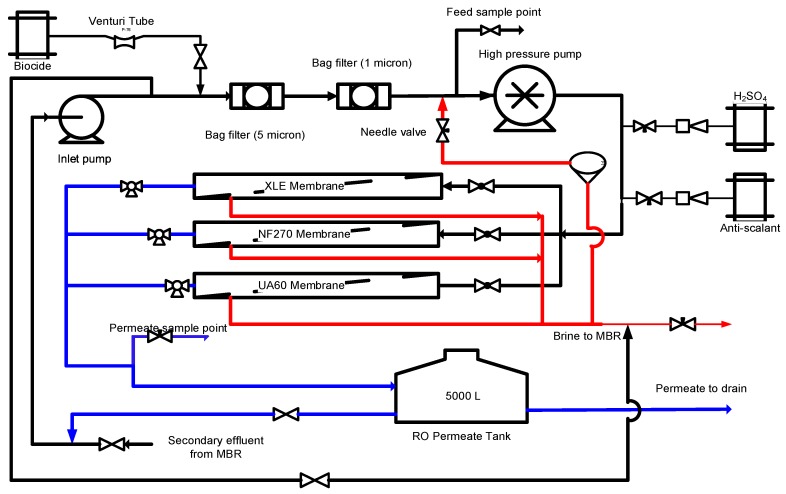
Process flow diagram of the ultrafiltration (UF)/nanofiltration (NF)/reverse osmosis (RO) pilot plant.

The contaminant removal efficiency was evaluated at a WWTW designated for possible agricultural, recreational, and potable reuse of wastewater effluents. This treatment plant consists of a full-scale membrane bioreactor (MBR) plant receiving wastewater from a densely populated residential area followed by UF/NF/RO pilot plant. The pilot plant consisted of three different thin film composite (TFC) polyamide (PA) membrane modules, in parallel, which was subjected to various experimental running conditions (Table 1). Secondary MBR effluent was used (Table 2) to feed into the pilot plant (Figure 1). Batch, 8 h, once through mode experimental runs were conducted on the pilot plant with individual membranes at any given time. Permeate ($C_{P}$) and feed ($C_{F}$) conductivities were used to evaluate the salt rejection (R) of the membrane as shown in Equation (1) [21]. Different operating conditions of flux and percentage recovery were used during the experimental runs. Table 1 shows a summary of the experimental conditions. The permeate flux (J) was calculated using the volume of permeate (V) collected through the active surface area of membrane (A) for a given period of time (Δt), as shown in Equation (2) [12].

(1) $$R=\;\left(1-\frac{C_{p}}{C_{f}}\right)\times 100$$

(2) $$J=\frac{V}{A\;\Delta T}$$

### 2.3. UF/NF/RO Membranes

High-pressure membranes examined in this project included the NF270 (Dow Chemical Co., Filmtec NF270-4040, Midland, MI, USA) polyamide TFC loose nanofiltration (NF) membranes, UA60 (TriSep, 4040-UA60-TSA, Goleta, CA, USA) piperazine-based TFC loose ultrafiltration (UF) membranes, and the XLE (Dow Chemical Co., Filmtec XLE-4040, Midland, MI, USA) polyamide TFC low-pressure reverse osmosis (RO) membrane. RO and NF membranes are considered thin-film composite comprising three layers: 0.2 μm polyamide, 40 mm polysulfone, and 120 mm polyester support web. The characteristics of these membranes are presented in Table 3.

### 2.4. Estrogenic and Androgenic Steroid Hormones

Target compounds selected for this study included natural steroidal hormones; an endogenous estrogen, 17β-estradiol (E_2_); and an endogenous androgen, testosterone (E). The physicochemical properties are presented in Table 4 [23], where the chemical structure shows that both E_2_ and T had two oxygen-containing functional groups, which took the form of primary or secondary alcohol or a ketone [24]. E_2_ and T had very low solubility in water. Their K_ow_ values suggest their hydrophobic nature and moderate-to-high binding to organic colloids and macromolecules in water.

### 2.5. Sample Collection and Solid Phase Extraction (SPE)

Sampling was carried out during May, June, and July as well as October, November, and December. The sampling points were (1) municipal wastewater raw-sewer (influent), (2) MBR influent, (3) MBR effluent, and (4) permeate of UF/NF/RO element. Grab samples were taken once weekly of both influent and effluent. To avoid frequent fluctuations in concentrations, each sample taken from the pilot plant was an 8 h composite sample taken for the duration of each experimental run.

All water samples were collected in amber glass bottles (2.5 L), covered with tin foil, placed on ice, kept in a cool box, and transported to the laboratory for testing. There was no contamination or contact with the plastic lid of the bottles. Once they reached the laboratory, the effluent samples were filtered through 1.0 μm pore size glass fibre filter paper (Whatman GF/B), then the filtrates were stored in a refrigerator at 4 °C, and solid-phase extraction (SPE) was performed within 48 h.

### 2.6. Enzyme-Linked Immunosorbent Assays (ELISAs)

17β-Estradiol (E_2_) and testosterone (T) concentrations were determined using enzyme-linked immunosorbent assay (ELISA) kits. Inter- and intra-assay variation for steroid hormone ELISAs are negligible, as shown by [10] who determined inter-assay variation at 5.6% (*n* = 3) and intra-assay variation between 0.6% and 2.5% (*n* = 3). Thus, the accuracy of the ELISAs reduces the need for expensive and time-consuming replication and provides for a rapid screen of several samples. All reagents required for the assays were supplied with the kits. E_2_ and T levels were determined in the C18 SPE extracts of water collected using commercially available ELISA kits (E_2_ and T, DRG International Inc., USA); according to the manufacturers’ instructions. Assay ranges of the kits are estradiol 9.7–2000 ng/L and testosterone 83–16,000 ng/L. The extracted samples in ethanol (1000× concentrated) were diluted (E_2_, 1/10; T, 2/10) in a 0.1% w/v human serum albumin and 0.9% NaCl solution and were assayed [10]. The diluted samples were then assayed using the kit, and the data obtained were plotted on the same graph as the standard curve to determine if the curves were parallel. The kits were assayed for intra-assay reproducibility by assaying replicates of the same sample on a single assay plate. The OD was determined at 450 nm using a plate reader. A standard curve was drawn using the reading obtained for the standards; the concentrations of the samples were read off this curve. Faul et al. (2014) [27] found that the effective lower level of quantification (LOQ) for each were reduced to 0.97 ng/L (E2) and 4.15 ng/L (T), respectively. Truter et al. (2015) [28] had the detection limits for E_2_ at 0.37ng/L, after a solvent blank correction.

### 2.7. In Vitro Recombinant Yeast Estrogen Screen (YES)

The recombinant yeast-based screen followed the protocol described by Sohoni and Sumpter (1998). *Saccharomyces cerevisiae* transfected with the human estrogen receptor (hER) gene and a plasmid containing an estrogen response element-linked *lac*-Z gene was used. Successful binding of ligands in the water samples (steroids) to the receptors in the yeast cells initiate the expression of the *lac*-Z reporter gene that encodes for the enzyme β-galactosidase in the assay. The β-galactosidase then metabolises chlorophenol red galactopyranoside (CPRG), which results in a colour change of the assay medium, indicating a dose-dependent activation of the ligands to bind to the estrogen receptor. The assay medium was prepared as described by [29]. The yeast was incubated in assay medium containing no CPRG for 48 h under 26 °C on an orbital shaker. The concentrated wastewater extracts (500×) were serially diluted and 10 µL was spiked into the 96-well sterile flat-bottomed plates with low evaporation lids (Costar, 3370, Sigma). The previously incubated yeast culture was then included into new assay medium containing CPRG at a concentration of approximately 8 × 105 cells/mL. The seeded assay medium was then added at 200 µL/well into the assay plate to provide a final concentration of the water extracts ranging from 50× to 1.56×. A concentration of 1× was depicted as an un-concentrated water sample. For the raw wastewater samples, serial dilutions of the samples were made with MeOH to obtain a concentration range of each sample ranging from 12.5× to 0.39× in the assay due to cytotoxicity observed in the 50× and 25× concentrated sample. For the effluent (permeate) water samples, serial dilutions of the samples were made with MeOH to obtain a concentration range of each sample ranging from 50× to 6.25× due to the lower observed estrogenicity in these samples compared to raw wastewater samples. All samples were analysed in triplicate in the same assay plate, and each assay was repeated twice. A standard curve for the steroid hormone 17β-estradiol (E_2_; CAS 50-28-2; Sigma) was included for each assay plate in 12 serial dilutions, ranging from 1.0 to 2700.0 ng/L. Blank wells were also included in each assay plate containing only assay medium without any hormone spike or water sample extracts. The assay plates were then allowed to incubate on a shaker for 72 h at 30 °C under dark conditions [30].

### 2.8. Statistical Analysis

All statistical analyses were performed using GraphPad Prism (v. 5.00) and Microsoft Excel 2010. The variation between individual samples was assessed using an unpaired *t*-test. For the determination of significant variation between sampling and membranes, a one-way analysis of variance (ANOVA) was performed. Significant variance was achieved with *p* < 0.05.

## 3. Discussion

### 3.1. ELISA Analysis of 17β-Estradiol (E_2_) and Testosterone (T)

**Figure 2:**
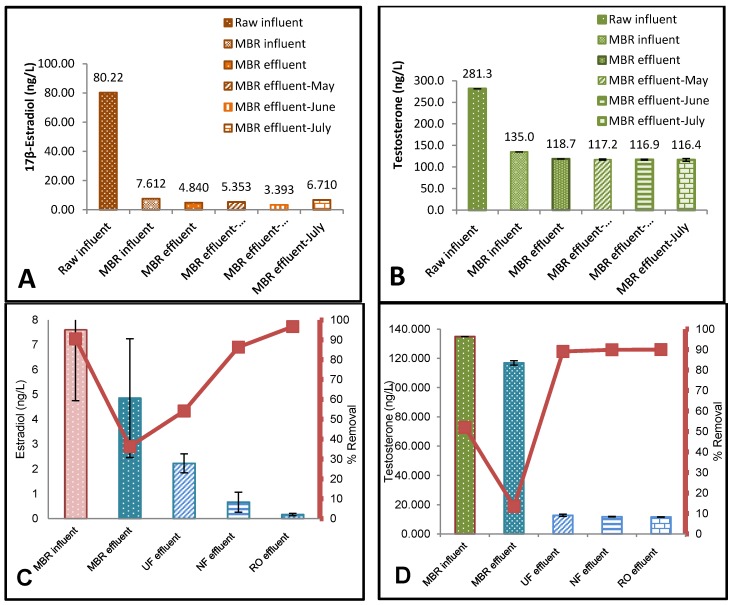
17β-Estradiol (E_2_) and testosterone (T) measured activity in collected samples from raw MWWTW and MBR influents as well as MBR effluents at six stages over three months. (**A**) shows the E_2_ ELISA analysis during May, June, and July. (**B**) shows the T ELISA analysis during the months of May, June, and July. (**C**) The mean of 17β-estradiol (E_2_) levels (ng/L) measured in water collected from influents at five stages within the MWWTW including MBR and RO feed and permeate during winter (May, June, and July) Error bars denote SD; *n* = 2. Error bars show maximum levels detected. (**D**) The mean of testosterone (T) levels (ng/L) measured in water collected from influents at five stages within the MWWTW including MBR and RO feed and permeate during winter (May, June, and July). Error bars denote SD; *n* = 2. Error bars show maximum levels detected.

Estradiol was detected in all influent samples analysed (Figure 2A). The highest E_2_ concentration was detected in the raw influent sample (80.22 ng/L), followed by the average MBR influent (7.61 ng/L), and effluent (4.84 ng/L). The MBR effluents (RO influent) for May, June, and July were 5.35, 3.39, and 6.71 ng/L, respectively. The highest concentrations of estradiol were found in the raw influent (Figure 2A), which was confirmed by Faul et al. (2013) [31] who measured E_2_ at the sewage inlet plant in Windhoek, Namibia at 78ng/L. A 91% removal of E_2_ was recorded in the anaerobic (anoxic) tank, where the raw influent was reduced from almost 80.22 to 7.61 ng/L. The lowest percentage removal (36%) (Figure 2C) was measured by the MBR aerobic (oxic) tank, where the MBR influent was reduced from 7.61 to 4.85 ng/L only. UF, NF, and RO had an expected percentage removal of 54%, 84%, and 97%. The change in MBR influent and effluent can be seen in Figure 2C, where the error was notable. This is an indicator confirming the fluctuation of the inlet streams. E_2_ was completely removed to below level of detection (LOD) for all XLE treatment processes with its removal efficiency of >93%. This agrees to previous results reported by Lee et al. (2008) [32] for secondary processes. The UF, NF, and RO effluents in sequence with MBR process were conserved to give very good efficiencies for the removal of E_2_ and T. Figure 2C shows that the E_2_ concentration for the effluents of MBR, UF, NF, and RO in sequence with MBR process had very low E_2_ concentrations of 4.85, 2.22, 0.66, and 0.16ng/L. NF and RO effluents had significantly reduced E_2_ concentrations compared with the influent at 7.61 ng/L (*p* = 0.007 at for UF, *p* = 0.00027 for NF, *p* = 0.00016 for RO; *α* = 0.05). This is consistent with a similar study of MBR/NF and MBR/RO membrane effluent rejection [33]. MBR is considered a relatively better treatment process for the removals of steroids compared to conventional activated sludge processes alone [34]. Likewise, micropollutants (MPs), such as E_2_ and T, can be removed by size exclusion and adsorption mechanisms using ultrafiltration (UF), nanofiltration (NF), and reverse osmosis (RO). The study by Lee et al. (2008) [32] showed that steroid hormones such as E_2_ can be removed by MBR/RO processes by 99%.

**Figure 3:**
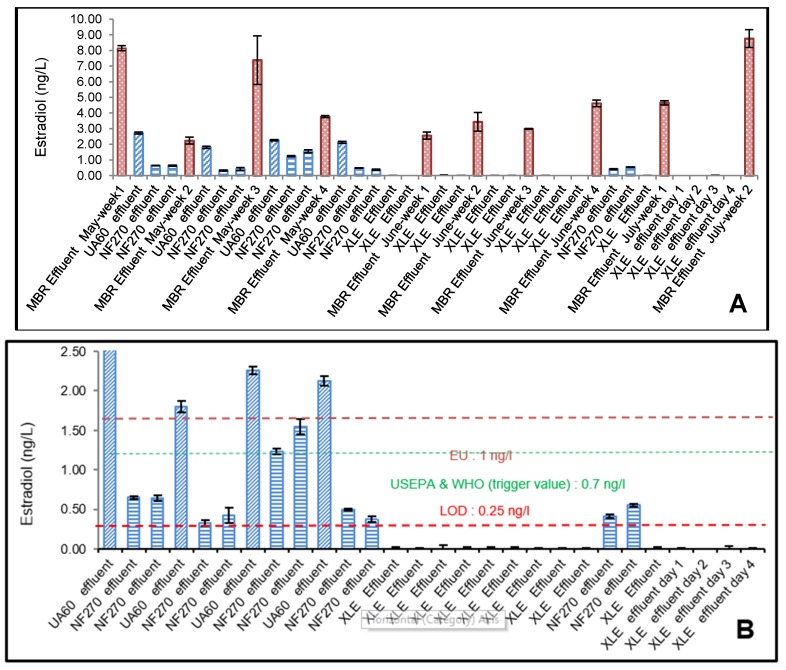

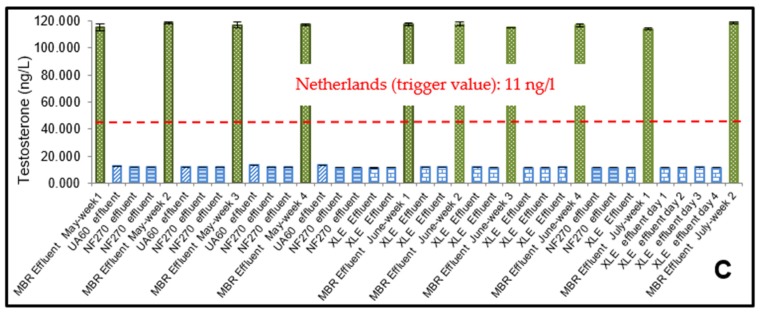
The mean ELISA of 17β-estradiol (E_2_) (ng/L) and testosterone (T) levels (ng/L). (**A**) shows the ELISA mean of 17β-estradiol (E_2_) levels (ng/L) measured in water collected from influent and effluents at various stages within the MWWW, including MBR and RO during winter (May, June, and July). Error bars denote SD; *n* = 2. Error bars show maximum levels detected. (**B**) shows the ELISA mean of 17β-estradiol (E_2_) levels (ng/L) measured of the effluents of the UF, NF, and RO membranes processes at various pilot plant conditions. Error bars denote SD; *n* = 2. Error bars show maximum levels detected (EU, 1 ng/L modulate fish production [41]; US EPA and WHO, 0.70 trigger value for drinking water [42], level of detection (LOD): 0.25 ng/L). (**C**) shows the ELISA mean of testosterone (T) levels (ng/L) measured in water collected from influent and effluents at various stages within the MWWW, including MBR and RO during winter (May, June, and July). Error bars denote SD; *n* = 2. Error bars show maximum levels detected.

Testosterone (T) was detected in all feed samples analysed (Figure 2B). The highest T concentration was detected in the raw influent sample (281.3 ng/L), followed by the average MBR influent (135.0 ng/L) and effluent (118.7 ng/L). The MBR effluents (RO influent) for May, June, and July were 117.2, 116.9, and 116.4 ng/L, respectively. Testosterone concentrations showed greater variation between the different samples with the highest as mentioned before with a raw influent concentration of 281.3 ng/L (Figure 2B) and lowest concentration after the MBR/RO process with an RO effluent (Figure 3C) of 11.4 ng/L. Testosterone levels measured corresponded well with those measured by Stalter et al. (2011) [35] in Switzerland and Germany (21 to 400 ng/L) and Manickum et al. (2014) [36] in South Africa (11 to 343 ng/L), whereas Fernandez et al. (2007) [37] in Canada and Chang et al.(2011) [38] in China, observed much lower concentrations (21 to 76.7 ng/L). However, the disparity in concentrations measured by Leusch et al. (2006) [39] was much more extreme (113 to 4300 ng/L). The mean MBR effluent before UF/NF/RO treatment was quite high, with an average value of 118ng/L (Figure 3C). Testosterone was almost completely removed in all the effluent samples after treatment with UF, NF, and RO membranes, with approximately 12 ng/L remaining (Figure 3C). This represents a removal efficiency of more than 90% as shown in Figure 2D, which is the same as recorded by Chang et al. (2011) [38] in China. The mean E_2_ concentration (Figure 2C) for the UF, NF, and RO effluents were 2.22, 0.66, and 0.16 ng/L, respectively. Figure 2D showed that the T concentration for the effluents of MBR, UF, NF, and RO in sequence with MBR process had lower T concentrations of 116.9, 12.75, 11.78, and 11.66ng/L, respectively. UF, NF, and RO effluents had significantly reduced T concentrations compared with the influent at 134.9 ng/L (*p* = 3.13 × 10^−20^ for UF, *p* = 4.67 × 10^−18^ for NF, *p* = 4.51 × 10^−18^ for RO; α = 0.05). The results were consistent with the previous study, indicating the downstream levels of the dams in Namibia with E_2_ and T concentrations of 7.2 and 19 ng/L, respectively [27]. The three processes, MBR/UF, MBR/NF, and MBR/RO, exhibited relatively similar T removal percentage (Figure 3C). In the UF/NF/RO stages following the MBR treatment, the removal percentage of all the T effluents were crowded into a very high but narrow range (e.g., 89% for UA60, 90% for both NF270 and XLE). According to Yangali-Quintanilla (2011) [40] the residual natural organic matter (NOM) increases the membrane removal potential by increasing the negative surface charge of the membrane, which therefore increases the electrostatic repulsion. It is also possible that these new conditions lead to contaminant rejection as a result of increased hydrophobic interactions with the membranes.

### 3.2. The Effect of Flux on Testosterone

The MBR/UF and MBR/NF systems were run at the same flux, but the MBR/RO system was run at two different fluxes (Table 1). An analysis of T removal rates by all systems implied that these filtration techniques can remove T to a very high extent (Figure 3C), although the results in all the applied fluxes were above the limit of quantification (LOQ) and on par with the Dutch drinking water trigger level of 11 ng/L [43]. Regardless of their high removal rates, however, T concentrations also exceeded the limit of detection (LOD). These results show that several molecules of T managed to penetrate the UF, NF, and RO membrane and, therefore, it was concluded that UF/NF/RO cannot serve as an absolute barrier to testosterone. Also, flux had no effect on testosterone removal. Sahar et al. (2011) [44] correspond with these findings in their investigation of the effect of three fluxes when removing MPs with CAS-UF/RO and MBR/RO systems.

### 3.3. YES Analysis of 17β-Estradiol (E_2_)

During the recombinant yeast estrogenicity bioassay screening (YES) test, no estrogenic activity was measured in the extraction control samples; thus, contamination of the cartridges during the extraction process can be excluded. Yeast growth was checked at an absorbance of 620 nm. Compared with the reference, none of the samples showed a decrease in cell density; therefore, no cytotoxicity was present. The sample was considered positive for estrogenic activity when three or more consecutive observations were above the level of detection (LOD) of the assay. The estrogenic activity (EEQs) of the samples was based on the EC_50_ value of the dose–response curves obtained for 17β-estradiol (E_2_) and the test sample. The level of detection (LOD) was calculated for each bioassay and experiment using the mean activity of the negative control and adding threefold its standard deviation. As the LODs varied between bioassays and membrane experimental runs, they were not all shown for the sake of clarity. However, in general, only results above the LODs were considered. In a few cases, such as estrogenic activity, lower activities were shown because of their ecotoxicological relevance (low effect threshold) and for comparing membrane effectivities.

Estrogenicity (binding to the human estrogen receptor (ER)) was detected in all feed samples analysed (Figure 2D). The raw influent had the highest proportion of estrogenic activity with 34.94 ng/L E_2_ equivalents (EEQs), followed by the average MBR influent (1.18 ng/L EEQs) and effluent (0.63 ng/L EEQs). The average MBR effluents (RO influent) for October, November, and December were 0.38, 0.74, and 0.44 ng/L EEQs, respectively.

**Figure 4:**
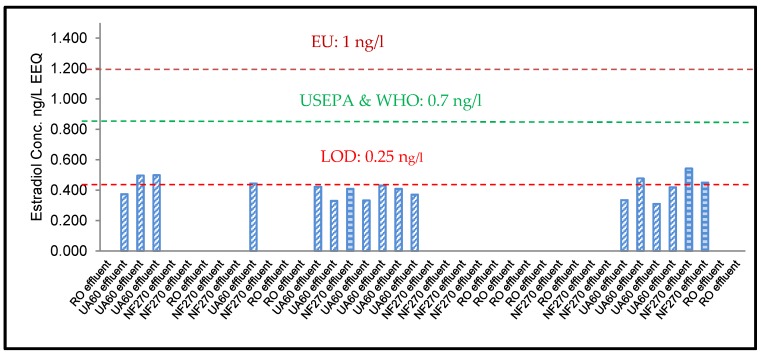
The YES of 17β-estradiol (E2) levels (ng/L estrogenic activity (EEQs)) measured in water collected from influent and effluents at various stages within the MWWW including MBR and RO, during October, November, and December (EU, 1 ng/L modulate fish production (Shappell et al., 2007); USEPA and WHO, 0.70 trigger value for drinking water [42], LOD: 0.25 ng/L).

Although EEQs (Figure 4) followed a similar trend as E_2_ concentrations (Figure 3A,B), the YES EEQs were slightly lower than E_2_ concentrations measured using ELISA. This is consistent with the previous study indicating higher E_2_ ELISA concentrations [36]. The YES and ELISA assay screening methods were the same, generating similar results for E_2_ as shown before, and thus no YAS (yeast androgen screening) for T was performed because the results were expected to be similar.

Variations in the pilot plant operating conditions in Table 1 (flux and pH) did not have any visible effect on the removal of E_2_ and T for the MBR/UF, MBR/NF, and MBR/RO processes. Rasak et al. (2007) [45] commented that the pH and pressure had a noticeable influence on the rejection of organic compounds. Rasak et al. (2007) [45] used a lab-scale cell with synthetic feed, whereas our study used a pilot plant with a real-time MBR feed-in once-through mode.

The physico-chemical properties of E_2_ and T are considered as responsible for influencing their rejection by UF/NF/RO membranes [5,46]. This can be observed in Figure 3A,B, where the MBR/RO treatment removed all the test samples of E_2_ below LOD < 0.25 ng/L, regardless of the specific operating plant conditions. Most of the MBR/NF treatment test samples were below LOD < 0.25ng/L. The rest were visibly between 0.33 and 0.65 ng/L. The MBR/UF treatment clearly showed poor removal of E_2_ with all four test samples between 2.12–2.72 ng/L. The retention of MPs in membrane separation processes depends on the characteristics of both the membrane and the pollutants. The hydrophobicity represented by the partitioning coefficient (*K*_ow_) of E_2_ and T as well as the adsorption, size exclusion, and charge repulsion, would have major influences on the rejection. The molecular size would be the overriding factor in the rejection by the UF/NF/RO membranes [13].

**Figure 5:**
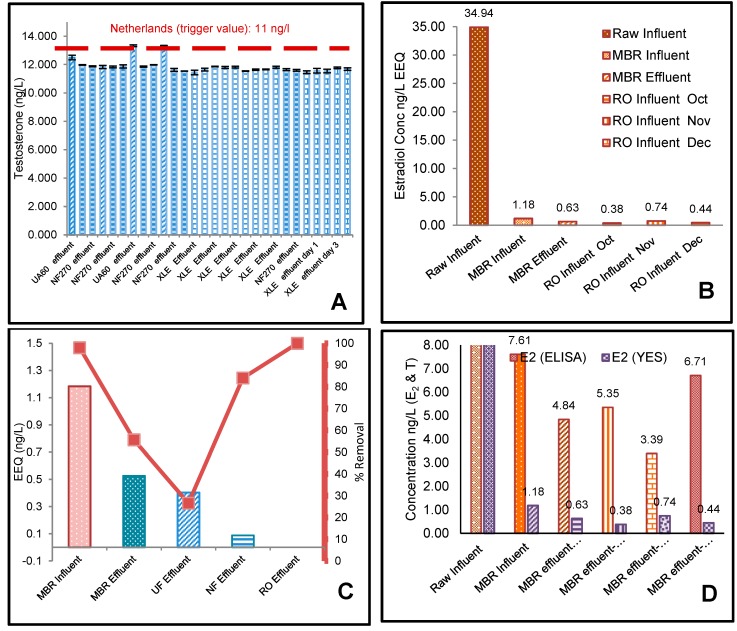
17β-Estradiol (E2) and testosterone (T) measured activity in collected samples from raw MWWTW and MBR influents as well as MBR effluents at six stages over three months, along with the percentage removal comparing the UF/NF/RO performance accordingly. (**A**) The ELISA mean of testosterone (T) levels (ng/L) measured of the effluents of the UF, NF, and RO membrane treatment at various pilot plant conditions. Error bars denote SD; *n* = 2. Error bars show maximum levels detected (Netherlands, 11ng/L trigger value for drinking water from Brand et al. (2013) [43]. (**B**) shows the E_2_ YES analysis during October, November, and December. (**C**) 17β-Estradiol (E_2_) levels (ng/L EEQs) measured in water collected from influents at five stages within the MWWTW including MBR and RO influent and effluent during summer (October, November, and December). (**D**) compares the ELISA and (YES) analysis results for E_2_.

Figure 5C shows that the EEQ values for the effluents of MBR, UF, NF, and RO in sequence with the MBR process had the very lowest estrogenic activities of 0.53, 0.43, and 0.088 ng-EEQ/L and LOD, respectively. NF and RO effluents had significantly reduced estrogenic activity compared with the influent at 1.18 ng-EEQ/L (*p* = 0.187 at for UF, *p* = 0.005 for NF, *p* = 0.007 for RO; *α* = 0.05). This is consistent with Ihara et al. (2014) [47],who showed highly reduced EEQ values in the effluent from advanced wastewater treatment processes with YES screening assays [32].

A 91% removal of testosterone (T) was recorded in the anaerobic (anoxic) tank, where the raw influent was reduced from almost 281.3 to 134.9 ng/L (Figure 2B,D). The lowest percentage removal (13%) was measured by the MBR aerobic (oxic) tank, where the MBR influent was reduced from 134.9 to 116.9 ng/L only. UF, NF, and RO had expected percentage removals of 89%, 90%, and 90%, respectively.

Testosterone was poorly removed by all treatment processes, with all effluent test samples measuring an average of 12ng/L (Figure 3C and Figure 5A). This could be due to the high dipole moment of the T compound. It is reported that a high dipole moment of a compound would lead to a decrease in the rejection by membranes [48]. A 98% removal of Estradiol was recorded in the anaerobic (anoxic) tank (Figure 5C), where the raw influent was reduced from almost 34.94 to 1.18 ng-EEQ/L (Figure 5B). The lowest percentage removal (55%) was measured by the MBR aerobic (oxic) tank, where the MBR influent was reduced from 1.18 to 0.53 ng-EEQ/L only. UF, NF, and RO had expected percentage removals of 27%, 84%, and 100% (Figure 5C).

## 4. Results

### 4.1. Effect of Membrane Properties on the Rejection 

The removal of E_2_ and T was due to the direct filtration by the UF/NF/RO membranes (Figure 2D, Figure 4 and Figure 5A–C). This was due to steric hindrance and their adsorption onto the polymeric membrane matrix. Adsorption can only contribute to short-term removal; as the feed is continuously filtered through the membrane, so membrane sites will be saturated with hydrophobic MPs. The charged and hydrophilic MPs did not adsorb to the polymeric membrane matrix and could be effectively removed by UF/NF/RO membranes via steric hindrance and electrostatic interaction mechanisms. Steric hindrance occurred because of the MW (E_2_: 272.38 g/mol; T: 288.42 g/mol), which was larger than the membrane pore size (MWCO) of the RO (<200 Da) and NF (400Da). The rejection increased as the MW of the MPs increased. This explains the poor performance of the NF membrane. The UA60, NF270, and XLE membranes used for this study were negatively charged. Thus, electrostatic interactions occurred between the accused MPs (E_2_ and T) and the negatively charged membrane surfaces, resulting in higher rejection compared to neutral solutes of a similar size. This result aligns with those of past studies [13,23,24,26].

The thin-film composite RO and NF membranes have about the same thickness, but the active layer of the NF is weaker. The diffusion governs the rate of E_2_ and T transport across the membrane through the active skin layer. Freger et al. (2002) [49] explained that water is lightly soluble in the polymer where the diffusion process between E_2_ and T takes place in a polymeric matrix saturated with small amounts of water. The authors also mentioned that the convective flow has only a small contribution to the transport of E_2_ and T across the membrane, but the presence of water is thought to play an essential role in facilitating the diffusion process [24]. This can be observed in Figure 4 and Figure 5C, where the NF/RO membrane treatment complemented MBR treatment very well, with the E_2_ and T being removed to below the level of detection (0.25ng/L) with 84% and 100% removal, respectively.

The combination of MBR with UF/NF/RO led to enhanced removal of MPs. The MBR/RO achieved higher removal efficiencies of E_2_ than MBR/NF and MBR/UF. Our observation is in good agreement with previous reports Nguyen et al. (2013) [23] and can be explained by the fact that NF270 and UA60 are loose membranes with a larger pore size and a higher permeability. This is also supported by the low conductivity rejection by the UA60 (10%) and NF270 membrane (41–49%) compared to the XLE membrane (93–95%) [23]. The molecular weights of E_2_ and T were considerably smaller than the pore size of the UF (UA60) membrane; therefore, most of the MPs could not be physically retained by size exclusion, as shown in Figure 4, where the E_2_ ng/L EEQ was above the LOD > 0.25 ng/L EEQ, thus having only a 26.5% removal (Figure 5C). Estradiol retention by the UF was due to adsorption to the membrane surface. This is clearly explained by Nghiem et al. (2004) [50] and Silva et al. (2012) [51], which consider the negative charge of the UF membrane and the dissociation constant of the MPs. McCallum et al. (2008) [52] investigated the adsorption and desorption processes occurring during the NF membrane filtration of E_2._ They explained that the adsorption of E_2_ onto the membrane and its desorption are dynamic processes, which meant that when the concentration of hormone in the feed solution is higher than in the membrane, adsorption will occur and the permeate concentration will increase until achieving an equilibrium; if the concentration in the feed solution is lower than the equilibrium concentration in the membrane, desorption will occur until a new equilibrium is reached.

Neale et al. (2009) [53] demonstrated that estradiol could interact with the bulk organic matter, including natural organic matter (NOM) surrogates such as humic acid. It adsorbs to the membranes through hydrophobic interaction, thus increasing the rejection. NF membranes retained estradiol due to both hydrophobic adsorption and size exclusion, whereas the UF membrane retained estradiol due to hydrophobic adsorption. This is well demonstrated in Figure 2D, where the NF (NF270) and UF (UA60) membranes achieved an 84% and 26.5% removal, respectively. This result aligns with those by authors Yoon et al. (2007) [54] and Silva et al. (2012) [51], where it can be concluded that estradiol retention by NF was significantly higher than that by UF. The removal rate of E_2_ and T using UF/NF/RO membranes is a function of the partitioning coefficient (log Kow) of E_2_ (4.01) and T (3.32), respectively. This was confirmed by Yoon et al. (2007) [54] who removed 25 MPs with UF and NF membranes, concluding that the retention increases with the increasing of the partitioning coefficient (log Kow). In the same research, a different retention trend was observed by Yoon et al. (2007) [54]—when MPs had a Log *K*ow of >2.8, they exhibited a percentage removal less than 40%, but when they had a Log *K*ow < 2.8, the percentage removal was more than 75%. This could be the possible reason why T concentration was not removed below the measure 12 ng/L (89 removal percentage) throughout all treatment samples, as shown in Figure 5A,B.

### 4.2. Risk Assessment of 17β-Estradiol (E_2_) and Testosterone (T)

Despite the moderate-to efficient removal of estrogenicity by the MBR/UF, MBR/NF, and MBR/RO treatment, the measured EEQ values still pose a potential adverse health risk. As conventional risk assessment approaches are focussed on acute or chronic toxicity endpoints, the use of predicted no-effect concentrations (PNEC) and no-observed effect concentrations (NOEC) are mostly incorporated to assess potential lethal toxicity in aquatic wildlife [55]. However, such an approach is focussed mainly on the toxicity of individual chemicals and, therefore, does not consider the complex mixture of interactions of environmental pollutants within a water system. The YES offers a viable option that indicates the net estrogenic potential of a water sample to modulate hormone receptor binding, with the estimated EEQs providing a semi-quantitative assessment of all compounds, which may mimic an estrogenic response similar to E_2_. It is, therefore, possible to compare such EEQ values to other toxicological studies [56].

The measured E_2_ and T concentrations were severely reduced in the effluent, although they were not removed completely during the UF treatment. This may still pose an environmental and health risk at very low nanograms per litre concentration levels. A multitude of studies has shown that the presence of natural steroid hormones in effluents has antagonistic effects on wildlife, including, among others, reduced fertility, abnormal development of male and female secondary sex characteristics, alteration in sex ratio, the feminisation of males, and change of behaviour [52]. Human and animal health is threatened when excess sewage effluent enters our water sources and effluent is used for irrigation application. In surface water, the effective lower LOQ for each was reduced to 0.97 ng/L for E_2_ and 4.15 ng/L for T [27,31]. Results of the ELISA for the male steroid hormone T are presented in Figure 3C and Figure 5A. All the effluent samples for the MBR/UF, MBR/NF, and MBR/RO were higher than the lower PNEC of the test, as well as the trigger value of 11 ng/L by Brand et al. (2013) [43]. Bandelj et al. (2006) [57] stated that androgenic substances in wastewater effluent could result in biological responses in animals, and the exposure of mosquitofish to androgenic substances in paper and pulp effluent has resulted in its masculinization [36].

The limit of detection (LOD) and limit of quantification (LOQ) were calculated as 3 x standard deviation of the negative control and 6 x standard deviation (SD) of the negative control, respectively [58]. The estrogenicity LOD was calculated as 0.25 ng/L (EEQ). Results of the ELISA and YES for the female steroid hormone E_2_ are presented in Figure 3A,B, Figure 4, respectively. All the E_2_ effluent samples with the MBR/UF, MBR/NF, and MBR/RO were lower than the lower LOQ of the test and were found to be less than the PNEC values for fish (1 ng E_2_/L) as proposed by Shappel et al. (2007) [41] and Faul et al. (2014) [27]. The PNECs are derived from the effect levels of the most sensitive test organism [59]. During in vivo vitellogenin (VTG) induction studies, the PNEC for E_2_ is appropriate for the application in risk assessment of aquatic organisms. The PNEC value for long-term exposure (i.e., >60 days) in water is 2 ng E_2_/L. Higher PNECs are recommended for short-term (i.e., a few days or weeks) exposure [60]. The authors summarise PNEC below 1ng/L as having no risk and above 10 ng/L as being high risk [61]. This is also confirmed by Shappell et al. (2007) [41], who stated a PNEC of 1 ng/L from the England and Wales Environmental Agency (2002). Stephen et al. (1985) [62] suggested a PNEC of 0.75 ng E_2_/L EEQ for protecting aquatic organisms from chronic and full-lifecycle exposures to E_2_. Caldwell et al. (2012) [60] and his colleagues recommended a slightly higher PNEC for E_2_ (2 ng E_2_/L), which was derived from investigating 21 in vivo NOECs. The European Union recommended a PNEC of (0.4 ng/L) E_2_ for protecting aquatic life [63]. From the perspective of safeguarding aquatic species rather than fishes only, environmental researchers argue that 0.75 ng E_2_/L may be more reasonable than 2 ng E_2_/L, and 0.75 ng E_2_/L may be more protective for aquatic organisms [63]. An estimated E_2_ trigger value of 0.7 ng/L [42] for drinking water standards and 0.4 ng/L EEQ [64] estrogenicity for long term fish exposure has been proposed, on top of which further monitoring should be considered to establish the identity and origin of the MPs [30]. To estimate the exact source of estrogenicity within environmental samples may prove difficult, which was the reason why chemical analysis of known estrogenic micro-pollutants was not considered during this study, and this has been confirmed by a previous study [55].

## 5. Conclusions

In this investigation, it was shown that RO and NF membrane processes exhibited exceptional removal rates (>95%) for E_2_ and T. The influent pH and flux did not influence the rejection of E_2_ and T, which was most likely ruled by adsorption, size exclusion, and diffusion simultaneously. Size exclusion was seemingly dominant, especially with NF and RO membranes. T, with a smaller partitioning coefficient (log Kow) value, was most likely adsorbed on the membranes and then passed through it to give a low rejection with all three membranes. It can be confirmed that the MBR/UF, MBR/NF, and MBR/RO comply with the USEPA, WHO, and EU trigger value PNEC as stipulated. It was found that RO showed higher removal percentages when compared with NF and UF. Consequently, domestic wastewater treated by MBR followed with NF or RO is adequate for the effective removal of natural steroid hormones.

## Figures and Tables

**Table 1:** Pilot plant operating conditions.

Parameters	Operating Conditions		
Membrane module	XLE	NF270	UA60
Recovery (%)	50; 75	75	75
Flux (L/m^2^hr^1^)	25; 30	30	30
pH	uncontrolled; 6.5	uncontrolled	uncontrolled

**Table 2:** The physicochemical characteristics of the membrane bioreactor (MBR) effluent.

Parameter	Units	Average MBR Effluent	Limit *
Electron conductivity (EC)	mS/m	56	75 *
pH		6.9	5.5–9.5 *
Chemical oxygen demand (COD)	mg/L	<20	75 *
Ammonium (NH_4_^2−^)	mg/L	<0.4	1.0 *
Phosphate (PO_4_)	mg/L	2.6	10 *
Nitrate (NO^3^)	mg/L	13	15 *
Chloride (Cl^−^)	mg/L	73	10 0 *
17β-Estradiol (E_2_)	ng/L	<5	-
Testosterone (T)	ng/L	120	-

* Department of Water and Forestry (DWAF) 2010 guideline [22].

**Table 3:** Properties of three membrane modules.

Membrane Component	Texture	Type	Rejection %	Effective Area (m^2^)	MWCO (Da)	Maximum Pressure (bar)	Maximum Temperature (°C)	Maximum Permeate Flowrate (m^3^/hr)
RO	TFC Polyamide	Filmtec XLE-4040	99% NaCl	8.1	<200	6.9	45	9.8
NF	TFC Polyamide	Filmtec NF270-4040	>97% MgSO4	7.6	400	4.8	45	9.5
UF	TFC Piperazine	TriSep 4040-UA60-TSA	80% MgSO4	8.2	1000	7.6	45	11.4

**Table 4:** Physiochemical properties of a selected estrogen and androgen compound.

Analytes	MW (g/mol)	Formula	CAS Number	Solubility (mg/L)	Dissociation Constant pK_a_	Classification	Partition. Coefficient (log K_ow_)	Chemical Structure
17β-Estradiol (E_2_)	272.38	C_18_H_24_O_2_	50-28-2	13	10.4	natural hormone (estrogen)	4.01	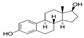
Testosterone (T)	288.42	C_19_H_28_O_2_	58-22-0	23.4	17.4	natural hormone (androgen)	3.32	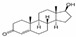

Physiochemical information was obtained from [25,26].
